# Association of accelerometer-measured physical activity and incident Parkinson’s disease: insights and future research directions

**DOI:** 10.1038/s41746-024-01154-0

**Published:** 2024-06-18

**Authors:** Li-Hua Chen, Tong Liu

**Affiliations:** 1 Institute of Pain Medicine and Special Environmental Medicine, Nantong University, Nantong, China; 2 Department of Nutrition and Food Hygiene, School of Public Health, Nantong University, Nantong, China

**Keywords:** Risk factors, Parkinson's disease

We congratulate Liu et al.^1^ for publishing the article in the *npj Digital Medicine*, an insightful article that has captured our keen interest. By addressing the limitations of self-report questionnaires and utilizing accelerometer-measured data from a substantial cohort of 96,422 participants in the UK Biobank, the research unveils insightful patterns. The identified L-shaped association for light physical activity (LPA) and moderate-to-vigorous physical activity (MVPA), along with the reversed L-shaped association for sedentary time, provides a nuanced understanding of PD risk. The consideration of exercise timing across different periods further enhances the comprehensive nature of the study. The findings substantially advance our understanding in this area. However, we believe that incorporating additional factors could provide even more robust insights. We have two major suggestions for future research that could build upon the valuable contributions of this study.

Firstly, sleep disorders should be considered. Sleep issues are tightly correlated to PD^2^, and deterioration of sleep quality has been demonstrated as markers of the prodromal phase of PD^3^. Previous investigations have indicated that specific sleep characteristics were linked to the incidence of PD^4–6^. Intervention in the prodromal phase might either delay cellular degeneration or slow its rate of progression. We noticed that this article did not consider the sleep issues. Whether physical activity could show a protective effect against PD in populations with the prodromal phase of PD remains unknown. Additional research is required to provide further evidence regarding the relationships between incident PD and objectively measured LPA and MVPA, especially within subgroups experiencing sleep-related issues. Concerning sleep disorders, while it may not have been the central focus of the original study, investigating their potential impact on PD risk could be pivotal for devising preventive strategies tailored to high-risk populations.

Secondly, the study did not comprehensively consider the confounding factors. Although the original study included some demographic and socioeconomic variables, they missed the important lifestyle factors, such as coffee and tea intake. Previous studies found that tea and coffee intake were inversely associated the PD^7–10^. Using directed acyclic graphs (DAGs) to illustrate potential biases and confounding factors would aid in better understanding the relationship between physical activity and PD.

These suggestions for further research that could complement and extend the current findings. By considering these additional factors, future studies could provide a more comprehensive understanding of the multifaceted relationships between physical activity, sleep, and PD risk, thereby elevating their clinical relevance.

## Reporting summary

Further information on research design is available in the Nature Research Reporting Summary linked to this article.

### Supplementary information

Reporting Summary
